# Early Left Parietal Activity Elicited by Direct Gaze: A High-Density EEG Study

**DOI:** 10.1371/journal.pone.0166430

**Published:** 2016-11-23

**Authors:** Nicolas Burra, Dirk Kerzel, Nathalie George

**Affiliations:** 1 Institut du Cerveau et de la Moelle Epinière, ICM, Social and Affective Neuroscience (SAN) Laboratory and Centre MEG-EEG, Paris, France; 2 Sorbonne Universités, UPMC Univ Paris 06, UMR_S 1127 and Centre MEG-EEG, Paris, France; 3 CNRS, UMR 7225 and Centre MEG-EEG, Paris, France; 4 Inserm, U 1127 and Centre MEG-EEG, Paris, France; 5 Faculté de Psychologie et des Sciences de l’Education, Université de Genève, Geneva, Switzerland; 6 ENS, Centre MEG-EEG, Paris, France; Centre National de la Recherche Scientifique, FRANCE

## Abstract

Gaze is one of the most important cues for human communication and social interaction. In particular, gaze contact is the most primary form of social contact and it is thought to capture attention. A very early-differentiated brain response to direct versus averted gaze has been hypothesized. Here, we used high-density electroencephalography to test this hypothesis. Topographical analysis allowed us to uncover a very early topographic modulation (40–80 ms) of event-related responses to faces with direct as compared to averted gaze. This modulation was obtained only in the condition where intact broadband faces–as opposed to high-pass or low-pas filtered faces–were presented. Source estimation indicated that this early modulation involved the posterior parietal region, encompassing the left precuneus and inferior parietal lobule. This supports the idea that it reflected an early orienting response to direct versus averted gaze. Accordingly, in a follow-up behavioural experiment, we found faster response times to the direct gaze than to the averted gaze broadband faces. In addition, classical evoked potential analysis showed that the N170 peak amplitude was larger for averted gaze than for direct gaze. Taken together, these results suggest that direct gaze may be detected at a very early processing stage, involving a parallel route to the ventral occipito-temporal route of face perceptual analysis.

## Introduction

The eye region is one of the most important face features in interpersonal interactions [1]. Gaze perception allows us to decipher the mental states of others: Gaze direction indicates the focus of another’s attention and it signals an individual’s upcoming goal [2–4]. In particular, direct gaze indicates attention directed at the perceiver; it is a salient stimulus, precursor to social interaction, with a threatening or positive significance depending on the facial expression (for reviews, [5, 6]).

It is therefore not surprising that direct gaze captures one’s attention. For instance, direct gaze has been shown to capture attention in visual search tasks [7–9] and to decrease the duration of face suppression during interocular suppression [10]. There is also evidence for the processing of eye contact when it is incidental to the task at hand as well as without focused attention or even in situations of continuous flash suppression where it is not consciously perceived [11, 12].

The attention capture by direct gaze suggests that direct–as compared to averted–gaze may elicit very early-differentiated neural responses during the processing of faces. Indeed, using an adapted dot-probe task with fearful, happy, or neutral faces serving as laterally presented cues, Pourtois and colleagues demonstrated that the capture of attention by fearful faces resulted in differentiated response to the probes appearing at the location of those faces (‘valid’ probes) -rather than at the opposite location of the screen (‘invalid’ probes)- between 40 and 80 ms [13]. This response was uncovered using topographical analysis of evoked potentials, which allows the detection of electric field differences independently of their strength [14]. Source localization further showed that this effect involved the left posterior parietal cortex, hence suggesting that it reflected an early, reflexive orienting response to the probes that appeared at the location of the fearful faces (see also [15] for a similar results in a Posner-like paradigm of attention orienting by fearful gaze).

No study to date looked for such an early effect (< 100ms) in relation with direct gaze processing. Studies using electroencephalography (EEG) have shown that the N170 component, which is typically elicited between 150 and 200ms and linked to the structural encoding of faces [16, 17], is modulated by gaze direction, with effects in different directions depending on task and stimuli. For instance, static pictures of faces under a frontal view generally yielded enhanced N170 for averted relative to direct gaze [18, 19] or no well-defined difference [20–23]. In contrast, enhanced N170 to gaze movement toward the perceiver, leading to direct gaze or not, was observed in explicit tasks of gaze direction judgment [18–20, 22, 24–27]. It therefore seems that the static versus moving nature of the stimuli, the head views included (frontal and/or deviated), as well as the explicit versus implicit nature of the task with regard to the face gaze can greatly influence the effect of gaze direction observed on the N170 in response to the faces. This might be explained by the fact that the processing of direct versus averted gaze may recruit different regions in posterior occipito-temporo-parietal regions, which may differentially impact and sum up at the level of the scalp N170, depending on the processes put at play according to task and stimulus parameters. In any case, one can retain that past studies have shown evidence for the neural coding of gaze direction in the time range of the N170. However, evidence for earlier (<100ms) differentiated brain responses to direct versus averted gaze is lacking.

In the present study, we aimed at investigating if the processing of faces with direct relative to averted gaze—presented at fixation—may be associated with very early differentiated neural responses. We used both conventional analysis of early ERP components (P1, N170) and topographical analysis of the event-related electric field patterns. The latter analysis might be particularly useful to reveal subtle differences in the topographical pattern of ERPs, which may not be picked up by the measurements of ERP components focused on a few selected electrodes [28, 29]. This has been shown previously by Pourtois et al. (2005) and in other studies on face perception (see, for example, [30, 31–35]). The rationale for such analysis is detailed by Pourtois, Delplanque, Michel, & Vuilleumier (2008) in the context of visual emotion processing. Our hypothesis was that using such analysis, we may be able to uncover very early (< 100 ms) differentiated brain responses to direct versus averted gaze, which could reflect early orienting responses to gaze. In addition, we used intact broadband faces and low-pass and high-pass filtered faces. Although spatial frequency filtering involves an important loss of information, we decided to include filtered faces to explore if early effects of direct versus averted gaze may be preferentially conveyed in the low spatial frequency range [36]. Indeed, low spatial frequencies are known to activate the magnocellular pathway that subtends fast, coarse visual processing while high-spatial frequencies activate preferentially the parvocellular pathway that is specialized in finer–but slower–processing [37–40]. In addition, it has been shown that some brain structures involved in processing information from the eye region, such as the amygdala [41–45]–although its role in processing gaze direction is debated [46], are sensitive to low spatial frequencies. It is therefore possible that the low spatial frequency faces might preferentially subtend very early responses to direct gaze. However, on the other hand, it is possible that only non-degraded, broadband faces may be able to trigger observable early flow of information in the visual cortex and therefore to reveal early differentiated brain responses to gaze direction.

## Methods and Methods

### Participants

Sixteen students of the University of Geneva without any neurological or psychiatric indications participated in this experiment for course credit (8 male/8 female; mean age ± SD = 21.6 ± 3.3). All participants had normal vision and were naïve as to the purpose of the experiment. All procedures were approved by the ethics committee of the University of Geneva (commission d'éthique de la Faculté de Psychologie et des Sciences de l'Éducation, Université de Genève) and written consent was obtained before the experiment started. One participant (1 male) was rejected because the signal was too noisy.

### Stimuli

**Fig 1:**
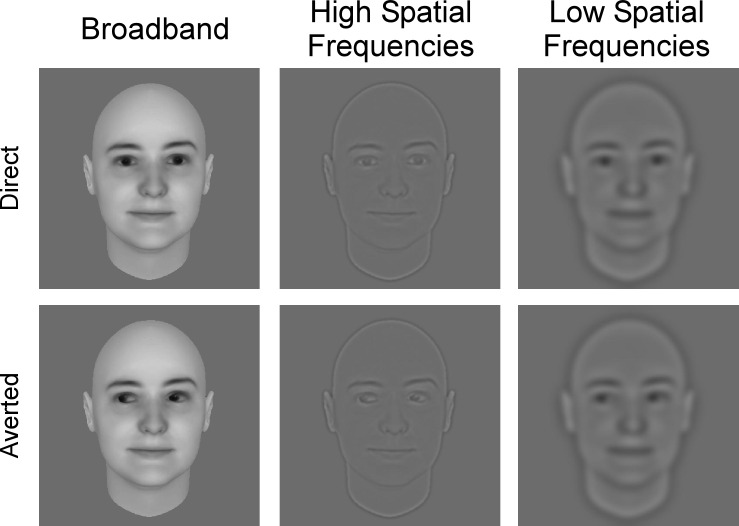
Example stimulus under each experimental condition of picture filtering (broadband / high spatial frequency / low spatial frequency) and gaze direction (direct / averted). These avatar faces were created with FaceGen Modeller 3.5 (see [45] for details).

The same stimuli as in Burra et al. (2013) were used (Fig 1). In brief, there were six different avatar face identities (3 male), each with direct and (right + left) averted gaze. Each picture was 512 pixels by 512 pixels (~6 degrees of visual angle). We used a wavelet decomposition to obtain the high (> 32 cycles/image) and low (< 6 cycles/image) spatial frequency versions of the faces [47].

### Procedure

Subjects were seated in a sound-attenuated room at 85 cm from a 17” LCD screen refreshed at 60 Hz. Each participant performed 8 blocks of 72 trials each, for a total of 576 trials (96 trials per condition). The order of conditions was randomized within every block. Each trial began with a fixation cross on a gray background for a random interval between 600 and 1600 ms. The face stimulus was then displayed and remained on the screen for 600 ms followed by an additional 200 ms gray background. Then, a response screen displaying the response mapping appeared and the participants were asked to report in the most accurate manner the gender of the face by a two-choice button press. After the participant’s response, the next trial was initiated, with an inter-trial interval (ITI) of 1000 ms. Participants were asked to blink only after their response or during the ITI. Each block took about 3 minutes to complete. Before the experiment, participants completed a 36 practice trials.

### EEG acquisition and preprocessing

A Biosemi ActiveTwo AD-Box amplifier system (Amsterdam, The Netherlands) with 128 active AG/AgCL electrodes. This system is referenced to common mode sense/driven right leg ground. The EEG signal was sampled at 1024 Hz. Then, using BrainVision Analyzer 2.1 (BrainProduct), we filtered our data off-line with high-pass and low-pass Butterworth zero phase filters (0.1 to 30 Hz with 24 db/oct.), thus using a high-pass filter that does not induce any distortion to the early components of the ERP [48]. When the electric signal of an electrode was too noisy during the entire recording, we used a spline interpolation technique (Order 4, Degree 10) in order to replace the electrode signal [49]. No more than 4% of the total number of electrodes was corrected. The signal was downsampled to 256 Hz and then recalculated against the average reference. A baseline correction (using the 200 ms of EEG signal before stimulus onset) was applied before artifact exclusion. Independent Component Analysis (ICA) was used to remove the eye blink artifacts. We excluded trials with any remaining activity larger than ± 80μV. On average, 22% (±11%) of the trials were rejected. EEG epochs were then averaged between 100 ms before and 350 ms after the onset of the face stimulus. The constant delay of 32ms introduced by the LCD screen (60hz) was recorder using a photodiode and then corrected so that the ERPs were precisely time-locked on the actual onset of the stimulus.

### Behavioral data analysis

In this experiment, the participants had to wait for a response screen to give their response, with emphasis on accuracy. Therefore reaction times were not informative and were not analyzed in detail (but see below additional behavioural experiment). As for response accuracy, the rate of correct answers in the gender task was analyzed using a two-way repeated-measures ANOVA with GAZE (Direct, Averted) and FREQUENCIES (Broadband [BB], High spatial frequency [HSF] and Low spatial frequency [LSF] faces) as within-subject factors.

### EEG data analysis

#### Analysis of ERP peak amplitude and latency

For the ERP analysis, we focused on the early visual evoked potentials, i.e. P1 and N170, which are respectively the maximal positive deflection between 90 and 130 ms and the maximal negative deflection between 130 and 190 ms, over posterior electrodes, in response to faces. We measured the peak amplitude and latency of the P1 and N170 under the different conditions of gaze direction and face filtering. We used the left and right occipito-temporal sites classically used to analyze these components [16, 17] with respect to the 128 electrodes system [50, 51]. The P1 peak was measured between the 100 and 140 ms and the N170 peak was measured between 140 and 160 ms over the pooled selected sites in the left and right hemispheres respectively. The amplitude of the P1 and N170 peaks were analyzed using repeated-measures analyses of variance (ANOVAs) with the HEMISPHERE (Left, Right), the FREQUENCIES (BB, HSF, and LSF), and the GAZE (Direct, Averted) as within-subject factors. When the comparisons had more than 1 degree of freedom, we used in addition Mauchly’s test to check for the assumption of sphericity. When the assumption was not violated, we report the original ANOVA; when it was violated, we used Greenhouse-Geisser correction and report the ANOVA with corrected degrees of freedom and the Greenhouse-Geisser epsilon (ε) value.

#### Global topographic dissimilarity analysis

In order to assess the topographic modulations of ERPs for the direct and averted gaze conditions, we first calculated the global dissimilarity between the electric field maps obtained in these two conditions for BB, LSF, and HSF faces. Dissimilarity analysis consists in the analysis of the raw spatiotemporal scalp distribution [14]; it indexes the differences in configuration between two electric fields, independently of their strength -i.e. global field power or GFP- (see [13] for a similar approach). This measure is a good complement to the spatial clustering analysis that we performed next (see below), because it gives indications about the periods of significant topographic modulation (for some additional details see [52, 53, 54]). Yet, it is not sufficient to determine if topographic differences are related to ERP configuration change (viz. microstate differences) or to shifts in latency between similar topographic electric field patterns. For this, we turned to micro-state segmentation.

#### Topographic analysis based on micro-state segmentation

We performed a spatial clustering analysis of the data using the Cartool software developed by Denis Brunet at the University of Geneva, Switzerland (http://brainmapping.unige.ch/cartool). Topographic analysis based on microstate segmentation allows the exploration of the difference in cortical generators between experimental conditions and it thus allows interpreting results with respect to their neurophysiological origins [53, 55–58]. This analysis is based on the observation that some electrical scalp topographies remain stable during a short period of time [59]; these topographies form the so-called micro-states. They can be identified using an automated spatial-clustering approach and allow summarizing ERP data by a limited number of field configurations, corresponding to the activation of different sets of cortical generators along time. An advantage of topographic ERP analysis over traditional waveform analysis is that it enables to bypass the issue of reference-dependent classic ERP analysis and the issue regarding a priori selection of electrodes or time period (see: [53]). This method has been used several times, with faces [31, 35, 60] or emotion [61, 62].

More precisely, using a topographical Atomize and Agglomerate Hierarchical Clustering (T-AHHC), we quantified the sequence of stable topographies, or maps, over the 350-ms time interval of the ERP at the group-averaged level of experimental conditions [63]. Topographical AAHC is a clustering approach that operates in a bottom-up manner. In a first step, this approach uses the maximal possible number of maps, i.e. the maximal number of time frames as the number of possible maps. Then, for each clustering computation, this number is reduced by aggregating the maps with the lowest global explained variance (GEV) together; the resulting maps are then re-submitted to clustering analysis, and the analysis is run iteratively until a defined criterion of clustering quality is reached. We used the Krzaniowski-Lai criterion [58] as an objective criterion of clustering quality and extracted the number of maps explaining the data most accurately.

The pattern of maps obtained can then be spatially correlated with the single-subject ERP maps, a stage referred as the “fitting” procedure that allows assessing quantitatively the presence of a certain map in the form of the GEV of this map for each participant. The spatial correlation between template maps and single-subject ERP topographies–GEV of the maps–was measured for every map that differed across the experimental conditions during a given time window, for each participant and under each experimental condition of gaze direction and picture filtering. This allowed us to quantify the representation of the template maps of interest across subjects and conditions. These measures of GEV across subjects and conditions were analyzed using an ANOVA with MAPS and EXPERIMENTAL CONDITIONS as within-subject factors.

### Source localization

Finally, using the distributed linear inverse solution and the Local Autoregressive Average Regularization Approach, named LAURA [64], we localized the cortical sources of the scalp ERPs in the time window where different patterns of electric fields were identified across our experimental conditions. For the lead field calculation, we used the Spherical Model with Anatomical Constraints method [65]. This model was applied to the 3005 solution points distributed in the grey matter of an anatomical MRI template, selected from a 6x6x6 mm grid equally distributed. It computed the lead field matrix using the known analytical solution for a spherical head model with three shells of different conductivity [66]. Based on the results of our topographic analyses of ERP patterns, we performed an ANOVA on the mean source amplitude in the time window of interest (41-80ms) in order to examine the differences in the sources for the different experimental conditions. We used a criterion for significance of p < .05, for at least 32 solution points of the brain volume, in order to control for multiple comparisons (see also [67, 68]).

### Additional behavioural experiment

Our EEG protocol allowed analysis of response accuracy only. In order to complement these data, we performed an additional behavioral experiment. Fifteen subjects (3 males, mean age ± SD = 22.7 ± 4.6 years) participated in this complementary experiment. The protocol was identical to that of the EEG experiment, except that the participants did not have to wait for a response screen to give their answer and they were asked to answer as quickly and as accurately as possible. Trials where the RT was faster than 200 ms or slower than 1500 ms were excluded from analysis. Medians RT of correct answers were computed and log transformed to normalize their distribution. The log-transformed median RT and the accuracy of gender categorization were analyzed using a two-way repeated-measures ANOVA with GAZE (Direct, Averted) and FREQUENCIES (BB, HSF and LSF) as within-subject factors.

## Results

### Behavioral data

The ANOVA performed on the accuracy of the subjects from the EEG experiment revealed only an effect of the spatial filtering of the faces, *F*(2,28) = 10.28, *p* < .001, η_p_^2^ = .48 with greater rate of correct gender responses for the broadband faces (mean rates of correct answers = .96, .90, .87, for BB, HSF, LSF pictures, respectively).

The additional behavioral experiment reproduced this finding, with greater rate of correct gender responses for the BB than the HSF and the LSF faces (mean rates of correct answers = .96, .86, .79, for BB, HSF, LSF pictures, respectively, *F*(2,28) = 13.17, *p* < .001, η_p_^2^ = .48).

**Fig 2:**
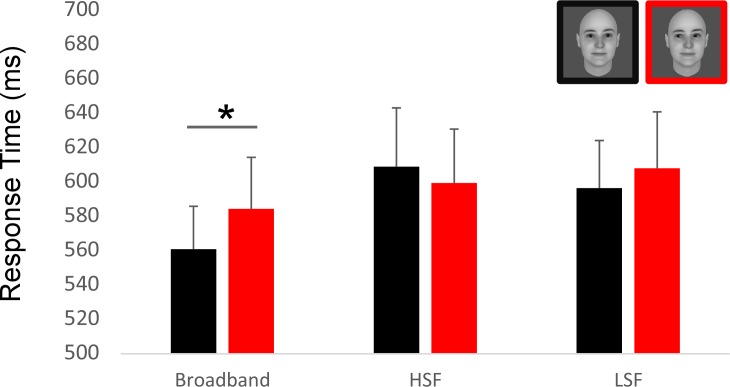
Result of the additional behavioral experiment. Using the same material, the same paradigm than the EEG experiment and the same number of participants (N = 15), we asked participants to answer as quickly as possible to the gender of each pictures. Critically, the direct gaze condition with broadband filtering is performed significantly faster than its averted counterpart.

Moreover, the analysis of RT in the additional behavioral experiment revealed also a main effect of the spatial filtering of the faces, with faster RT for the broadband faces (median RT = 572 ms, 604 ms, 602 ms, for BB, HSF, LSF pictures, respectively; *F*(2,28) = 8.65, *p* < .001, η_p_^2^ = .38). In addition, the RT was faster for direct than averted gaze for the broadband faces (mean RT for direct gaze = 560ms, for averted gaze = 584ms, *t*(14) = 2.38, *p* = .036, Cohen's *d =* 0.21) (Fig 2). The gaze direction effect was not significant for the LSF (mean RT for direct gaze = 596ms, for averted gaze = 608 ms; *t*<1) and the HSF faces (mean RT for direct gaze = 596ms, for averted gaze = 608 ms; *t*<1).

Altogether these results indicated that the non-degraded, broadband faces yielded faster and more accurate processing than the filtered faces, allowing to reveal the impact on gaze on the speed of face processing, with faster responses to the faces with direct than averted gaze.

### ERP peak amplitude and latency analysis

#### P1

There was neither any effect of GAZE nor any interaction between GAZE and FREQUENCIES on the P1 peak amplitude and latency.

The only effects observed on the amplitude and latency of the P1 component were main effects of FREQUENCIES. There was a main effect of FREQUENCIES on the P1 peak latency, *F*(2,28) = 77.94, *p* < .0001, ηp2 = .84, with a delayed latency for the HSF pictures (136 ± 1.6 ms) as compared to the LSF pictures (126 ± 2.6 ms) and for the LSF pictures as compared to the BB pictures (110 ± 2.8 ms). Post-hoc Bonferroni-corrected tests indicated that all three conditions were significantly different from each other (all *ps* < .001).

There was also a main effect of FREQUENCIES on P1 peak amplitude, *F*(1.13,15.89) = 20.95, ε = 0.56, *p* < .021, ηp2 = .30, with greater amplitude of the P1 for the filtered faces (HSF: 5.7 ± 0.69 μV, LSF: 5.34 ± 0.58 μV) than for the BB faces (4.9 ± 0.56 μV). Post-hoc Bonferroni-corrected tests confirmed that the BB condition was significantly different from both filtered conditions (LSF and HSF), *p* < .05.

#### N170

**Fig 3:**
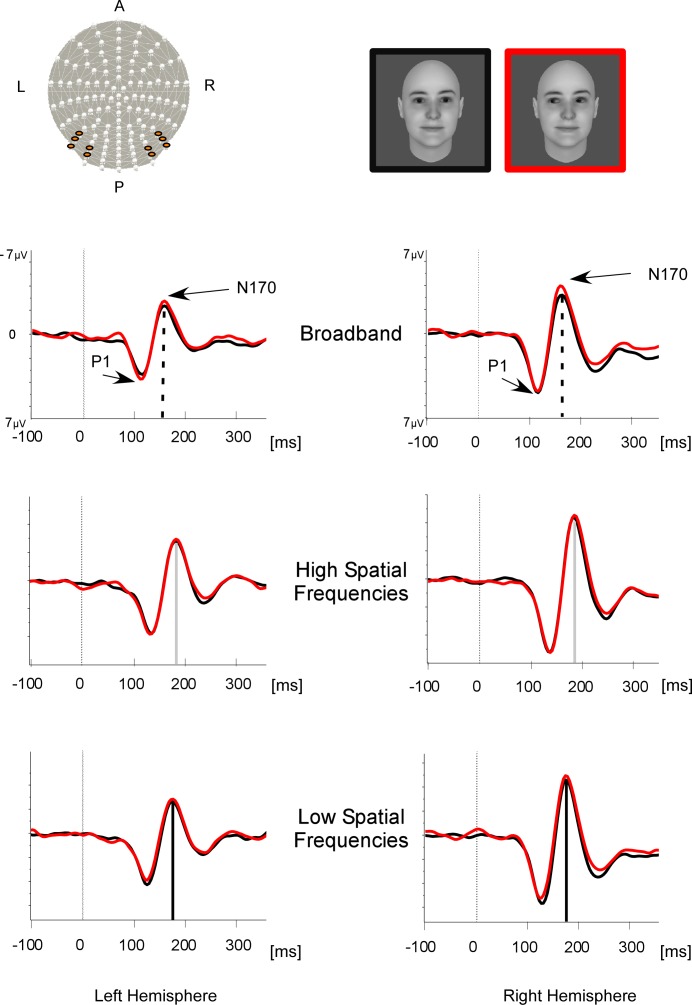
ERP analysis. Grand mean ERPs from the pooled left and right occipito-temporal electrodes (highlighted on the cap at the top left) are shown for the broadband (top row), high spatial frequency (middle row), low spatial frequency (bottom row) faces with direct gaze (in black) and averted gaze (in red). Both P1 and N170 peak latencies were delayed with filtered pictures. The dashed lines represent the peak latency of the N170 under each picture filtering condition.

There was a main effect of GAZE on N170 amplitude, *F*(1,14) = 7.91, *p* < .05, η_p_^2^ = .36 with statistically greater N170 for faces with averted gaze (-4.61 ± 0.92 μV) than for faces with direct gaze (-4.28 ± 0.93 μV) (Fig 3).

Furthermore, FREQUENCIES affected the N170 latency and amplitude. There was a main effect of FREQUENCIES on N170 latency, *F*(1, 36, 19.08) = 83.76, ε = 0.68, *p* < .0001, ηp2 = .86, with delayed N170 for the HSF faces (185 ± 2 ms) as compared to the LSF faces (175 ± 2 ms) and for the LSF faces as compared to the BB faces (163 ± 3 ms). Post-hoc Bonferroni-corrected tests indicated that all three conditions were significantly different from each other (all ps < .001). There was also a main effect of FREQUENCIES on N170 amplitude, *F*(2, 28) = 8.06, *p* < .002, ηp2 = .36, with greater N170 amplitude for the filtered pictures (HSF: -5.1 ± 1 μV, LSF: -4.42 ± 0.87 μV) than for the BB pictures (-3.8 ± 0.95 μV). Post-hoc Bonferroni-corrected tests indicating that this difference reached significance for HSF vs. BB faces (*p* < .05).

### Global topographic dissimilarity analysis

In order to test for very early topographical differences in event-related responses, we used a data-driven approach based first on the analysis of global dissimilarity between topographical maps at each time point and second on the segmentation of the data in micro-states (see [62] for a similar approach).

**Fig 4:**
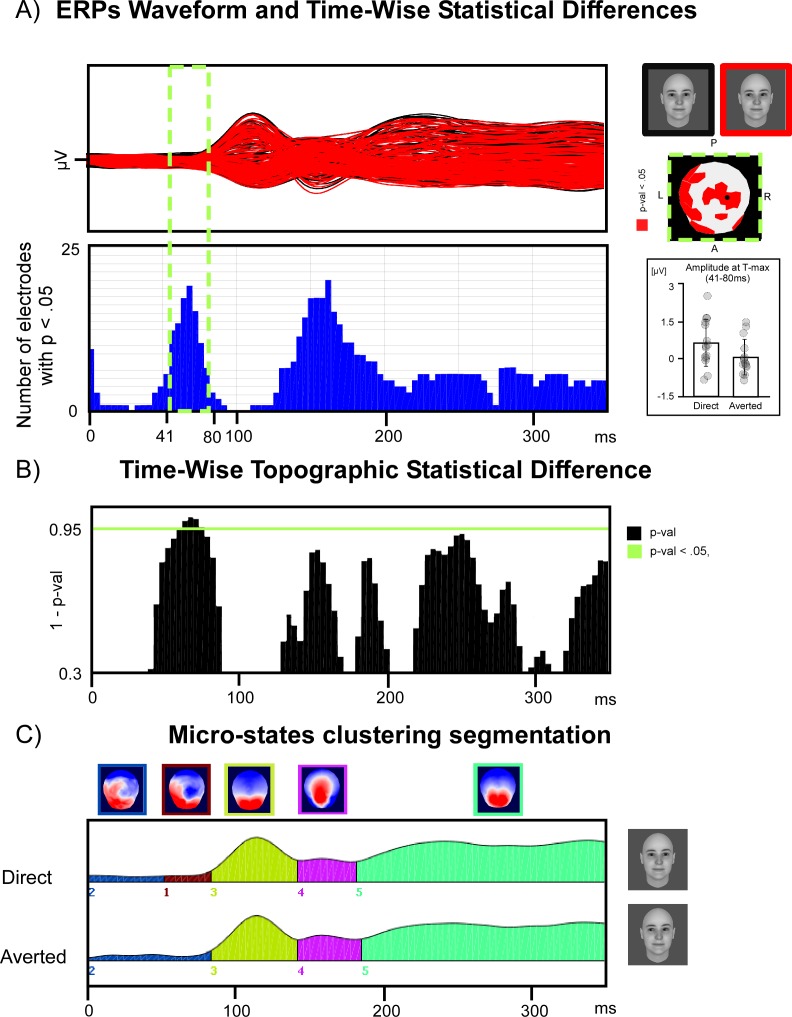
Early differences between topographical ERP patterns in response to direct and averted gaze, in the broadband (BB) picture condition. A) Overview of ERPs in response to direct and averted gaze BB pictures and sample-by-sample t-test of ERP differences between these two conditions. The *upper graph* represents the overlay of grand mean ERP waveforms obtained on every electrode in response to direct and averted gaze BB pictures. The *lower graph* represents the number of electrodes for which the p value of the exploratory t-test of ERP differences between direct and averted gaze conditions (performed on each electrode and each time point) reached significance (at an uncorrected threshold of *p* < .05). The time window delimited by a dashed green rectangle represents the 41–80 ms time window in which different topographical maps were identified for the direct and averted gaze BB picture conditions (see part C of this figure and text). On the right of the graph, a topographical map shows the electrodes that reached the .05 threshold p-value and a bar plot illustrates the early effect showing a scatterplot of the individual participants’ mean amplitude values between 41 and 80 ms for direct and averted gaze at the electrode of maximal t-value (represented by a black dot on topographical map). This showed that the early effect was present in every participant but one. B) Global topographic dissimilarity analysis. The graph represents the statistical level of the global dissimilarity index between the ERP scalp distributions for direct and averted gaze, in the BB picture condition. C) Micro-state segmentation. The five stable topographical maps obtained using the microstate segmentation procedure are represented above the Global Field Power (GFP) for direct and averted gaze BB pictures. The areas under the curves of the GFP are coloured to represent the period of time where each map was stable. Between 41 and 80 ms, distinct maps (maps 1 and 2) were identified for the direct and averted gaze conditions.

The global topographic dissimilarity analysis revealed a very early differentiation of the ERP topographic patterns in response to direct and averted gaze for the BB faces. This topographic dissimilarity was maximal at 70 ms, reaching significance between 68 and 76 ms (*p* < .05) (see Fig 4B). This very early topography modulation was also seen as a trend for the LSF pictures and was not significant for the HSF pictures (see S1 and S2 Figs).

Additional analysis performed by running classical t-tests at each electrode and time point confirmed the very early effect found and suggested that the topography dissimilarity corresponded to ERP differences between direct and averted gaze conditions over posterior electrodes (see Fig 4A).

We then turned to micro-state segmentation to test formally for the differences in topographical patterns between the direct and averted gaze conditions in BB faces.

### Micro-state segmentation

**Fig 5:**
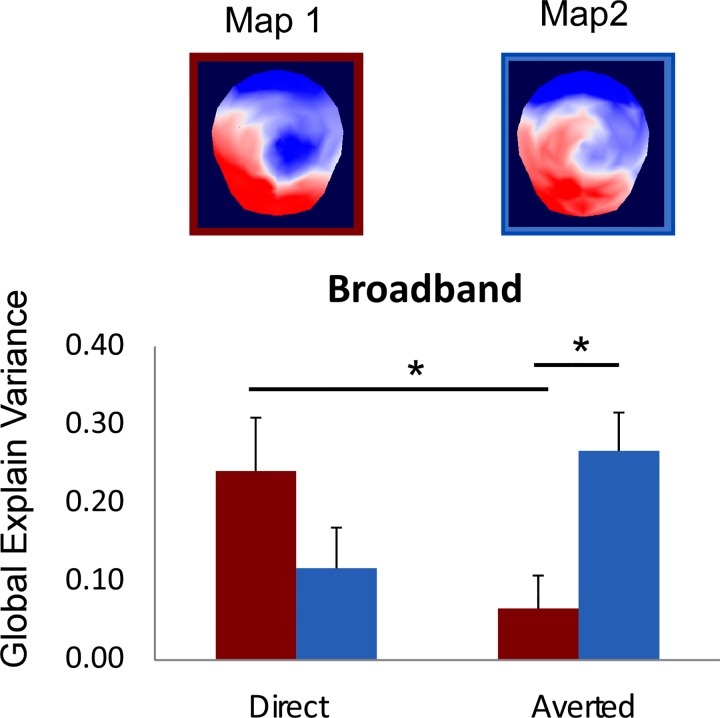
Result of the fitting procedure for the two early maps (Map1 / Map 2) identified in the 41–80 ms time window. There was a significant interaction between MAP and GAZE in the BB picture condition. The fitting procedure showed that the Map 1 explained more variance in the direct than in the averted gaze condition and that Map 2 explained better the averted gaze condition than Map 1 did. In addition, post-hoc t-test showed that Map 1 explained better the direct gaze than the averted gaze condition, *t*(14) = 3.33, *p* < .005, and that Map 2 explained better the averted gaze condition than Map 1 did, *t*(14) = 2.73, *p* < .016. Since normality condition was not verified for this explained variance measure, we ran additional non-parametrical Wilcoxon signed-rank tests; this confirmed the results reported with Zs < -2.04, *ps* < .05.

We performed spatial clustering on the grand average ERPs in order to look for a possible early effect of gaze for the BB face pictures resulting in different scalp topographies. The microstate segmentation found 5 different maps of stable ERP topographies that explained 94.6% of the ERP data. Most interestingly, this analysis revealed a difference in the early maps identified for the direct and the averted gaze conditions of BB pictures between 41 and 80 ms. Later maps were similar across conditions and corresponded to the P1, N170, and P300 components of the ERP (see Fig 4C). In order to evaluate how much the early “prototypical” maps identified on the grand averaged data explains the individual scalp topographies obtained in each participant, we computed the GEV for the first two maps obtained in response to the direct and averted gaze in each spatial frequency range for each participant, and we performed an ANOVA on these GEV values with MAPS (map 1 / map 2, as numbered in Figs 4C and 5), GAZE (direct / averted) and FREQUENCIES (BB / LSF / HSF) as within-subject factors This analysis revealed a three-way interaction between MAPS, GAZE, and FREQUENCIES, *F*(2,28) *=* 7.72, *p <* .002, η_p_^2^ = .35. This interaction reflected the MAP-by-GAZE interaction that was observed only for the BB pictures, *F*(1,14) = 8.85, *p* < .01, η_p_^2^ = .38 (see Fig 4). In other terms, this analysis confirmed that the 2 maps identified between 41 and 80 ms were differentially represented in the direct and the averted gaze BB picture conditions across participants.

### Source localization

**Fig 6:**
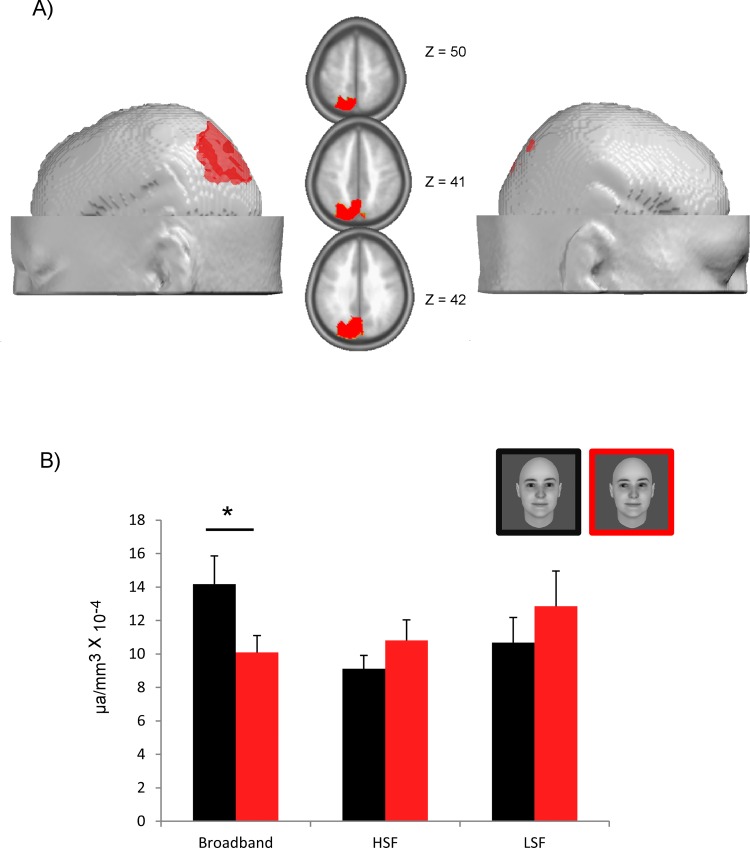
Source localization in the 41–80 ms time window. A) Result of the node-wise ANOVA performed on source estimation in the 41–80 ms time window. The nodes showing a significant interaction between FREQUENCIES and GAZE (*p* < .05 over a minimum of 8ms with a cluster size > 32 solution points) are represented in red over left and right lateral views and horizontal sections of the template brain. B) Bar plot of the estimated source activity over the left parietal cluster identified in A, under each condition of picture filtering (Broadband, HSF, LSF) and gaze direction (direct gaze in black, averted gaze in red), showing that the left parietal region was significantly more activated for direct than averted gaze in the broadband face condition.

In order to identify the candidate regions underpinning the early topographic difference between direct and averted gaze conditions, LAURA distributed source estimations were calculated over the 41–80 ms time period. We submitted the mean source amplitude estimations obtained in this time window to an ANOVA with FREQUENCIES and GAZE as within-subject factors. This revealed a significant interaction between FREQUENCIES and GAZE, with a maximum peak localized in the left posterior parietal lobe region [Talairach, -9,-82, 41], close to the left precuneus and extending into the inferior parietal lobule, *F*(2,28) = 4.22, *p* < .025, ηp2 = .23 (Fig 6A). Post-hoc t-test performed on the source points extracted from this region revealed that direct gaze activated this region more strongly (1.42 ± .103 nV/mm3) than averted gaze (1.01 ± .118 nV/mm3) in the BB picture condition, t(14) = 3.75, p < .02. This effect was not significant for the LSF and HSF pictures (Fig 6B).

## Discussion

In this experiment, we investigated the neural responses to faces with direct and averted gaze using high-density EEG and topographical analysis of event-related responses in order to examine if very early differentiated neural responses to direct versus averted gaze could be observed. While classical evoked potential analysis revealed an effect of gaze direction only in the form of enhanced N170 for averted relative to direct gaze, the topographical analysis revealed differentiated ERP topographies to direct and averted gaze as early as between 41 and 80 ms. Source localization further suggested that this early differentiated response involved the left posterior parietal cortex, mainly in the precuneus region. These results were obtained in the condition of BB face picture presentation, while LSF and HSF picture conditions did not allow us to differentiate these early brain response to direct versus averted gaze. In addition, a separate behavioural experiment showed faster gender categorization responses for direct than averted gaze in the broadband condition only.

Considering the importance of gaze contact in interpersonal interactions and the available evidence that direct gaze elicits attention capture, the present study aimed at testing if very early (< 100 ms) brain responses to direct versus averted gaze may be detected. To uncover such effects, we used a technique for topographical analysis of the scalp electrical activity that is able to reveal subtle differences in the topographical patterns of evoked electrical activities [14, 52, 53, 56, 57, 62]. This technique enabled us to investigate global activity patterns at the scalp level. With this approach, we uncovered a very early global dissimilarity between direct and averted gaze, peaking around 70 ms. This very early difference in the electric field patterns in response to direct and averted gaze was demonstrated with broadband (BB) pictures only. It is possible that the filtered pictures elicited smaller responses or responses of lower signal-to-noise ratio, particularly in the very early time range of the first flow of activation in the cortex, obscuring any early effect in the HSF and LSF picture conditions. As for BB pictures, microstate segmentation further confirmed the difference in the scalp topography of electrical activities elicited by direct and averted gaze, with the identification of two distinct topographic maps or microstates between 41 and 80 ms (similar as for fearful faces, as demonstrated by, [69, 70]).

Source localization indicated that this topographical difference reflected differentiated activation to direct gaze as compared to averted gaze BB pictures in the left posterior parietal cortex region, encompassing the precuneus and the inferior parietal lobule. Some studies have suggested that the parietal lobule and the precuneus work together in directing attention in space during both covert and overt attention orienting [71]. In the right hemisphere, the posterior parietal cortex has been associated with the spatial attention shifts triggered by averted gaze perception [3, 72]. Moreover, both the right and left precuneus were involved in saccade and pointing tasks, and additionally, the left precuneus showed greater activation specific to target detection when no eye movement was involved [73]. The role of these regions extends beyond the spatial aspects of attention. In a task where subjects paid attention to a specific central target, the precuneus region was more activated when attention switched between different target features, as compared with a condition of sustained attention to a specific target feature. This highlighted the role of these brain regions in non-spatial shifts of attention [74]. The posterior parietal region would also be involved in the early attention orienting toward relevant stimuli [75], defined as the process that give rise to an attentional biasing signal leading to initiate amplification and/or prioritization of the processing of the incoming stimulus. This process would take place at pre-attentive stage, allowing allocation of central processing resources to relevant or novel stimuli [76, 77]. It could be driven by attention capture elicited by the salient stimulus formed by direct gaze as well as by a ‘natural’ bias toward direct gaze processing, related to its biological and social significance. Interestingly, the region of the precuneus is at the interface between the first visual flow of information (viz. bottom-up information) and high-level cognitive processes (viz. top-down influences). Resting-state functional connectivity has revealed that the precuneus region is connected with the primary visual regions and with some more cognitive regions such as the inferior parietal lobule, the angular gyrus, and some prefrontal areas, close to the frontal-eyed field [78, 79]. More, generally, the parietal cortex has been reported to be involved in the integration between top-down and bottom-up attention [80–84]. Thus, we suggest that the very early differential activation of the precuneus and inferior parietal lobule region that we uncovered reflects a very early attention capture response associated with direct gaze perception, in relation with its biological relevance. In agreement with this view, in the additional behavioural experiment that we conducted, we found faster RT to the faces with direct than averted gaze in the gender categorization task (for similar results, see: [85]).

Our study is the first to report early posterior parietal activity associated with direct gaze perception. However, other relevant biological stimuli have been reported to activate this region, such as facial expressions [86] or biological motion for instance [87]. Hence, this posterior parietal region appears to drive the rapid, automatic orienting response to highly relevant stimuli (e.g. aversive), which may occur even outside of consciousness. When testing hemianopic patients, the presentation of a visual stimulus associated at a prior stage with a threatening sound, in the blind field of the patients, induced an enhancement of left parietal cortex activation, in spite of a lack of stimulus awareness [88]. Furthermore, fearful faces presented without awareness activated the left posterior parietal region, too [89]. Thus, our results are consistent with a prominent role of the posterior parietal region encompassing the precuneus in the reflexive attention capture elicited by the highly biologically relevant and salient stimulus formed by direct gaze in humans [90]. Compared to other species, humans have a highly salient eye region [1], which may facilitate the early detection of gaze direction by the human brain. Our study suggests that gaze detection may take place very early on during face processing, leading to attention capture by direct gaze.

The early time window of our effect raises the question of its cerebral origin. Such early brain response may reflect fast visual pathways, involving either rapid feedforward flow of cortical information through V1 or subcortical-to-extrastriate-cortical pathway bypassing V1 [91, 92]. In this line, Foxe et al., [93] brought evidence for the fact that only the early part of C1 would reflect pure V1 activation; while its late part would reflect distributed activation extending into extrastriate, parieto-temporp-frontal regions. Moreover, in macaques, onset latencies of 26.3 ms and 25.8 ms were found in V1 and IP respectively ([94], see also, [95]). In the pulvinar thalamic nucleus, visual response latency was comprised between 60 and 80 ms after stimulus onset [96], coinciding with the response latency in early visual cortical areas V1 and V2 [96, 97]. Interestingly, the pulvinar is implicated in processing salient face information, as emotional expressions of human faces activate neurons in the monkey pulvinar [98]. Moreover, it projects directly to the parietal lobe [99–102]. Such functional and structural connection between pulvinar and parietal cortex is a likely candidate pathway of the early effect found here.

We also performed classical ERP parameter analysis of the P1 and the N170 components, which are typical signatures of visual stimulus processing and face perception: The P1 has been claimed to be related to extra-striate visual activities [103–105], while the N170 has been linked to the structural encoding of faces [16, 17].

The P1 and N170 components were both affected by the face picture filtering, with delayed P1 and N170 peak latency for HSF pictures relative to LSF pictures and for LSF pictures relative to BB pictures as has already been reported in previous experiments [106, 107]. This suggests that spatial filtering results in a slowing of the visual processing of the pictures, probably because picture degradation through filtering slows the accumulation of evidence [108] at the early stage of visual processing, hence inducing a delayed peak latency of the P1, also reverberated on the N170 latency. This interpretation is also consistent with the increased P1 and N170 amplitudes obtained for the filtered as compared to the broadband pictures, which would reflect the need for more evidence accumulation [108] for the degraded (filtered) relative to the intact (broadband) picture processing. Such amplitude effects, particularly on the N170 for HSF, might be also related to our gender task, which required extraction of stimulus features [109].

Moreover, we found a small but statistically significant effect of gaze on the N170 peak amplitude, with larger N170 for the averted than the direct gaze conditions. Effects of gaze direction on N170 amplitude have already been reported but with discrepancies in the effect of observed, the N170 being either enhanced or reduced for averted relative to averted gaze across studies [18, 27, 110]. The origin of these discrepancies might be related to several factors, such as stimulus (full faces vs. deviated faces), presentation type (static vs. dynamic), and task differences across studies (gender task vs. gaze discrimination), as recently demonstrated by Latinus et al. (2015). It is likely that the processing of gaze direction, head orientation, and gaze motion recruit different close-by areas in posterior occipito-temporo-parietal regions, which may differentially impact and sum up at the level of the scalp N170, depending on the stimulus and task at hand. Our result is in line with previous studies that used static faces under frontal view and found enhanced N170 for faces displaying averted as compared to direct gaze ([18, 19], see also [111]).

We obtained distinct effects of gaze direction in the 40–80 ms and in the N170 time ranges, with involvement of posterior parietal cortex in response to direct as compared to averted gaze between 40 and 80 ms and increased N170 to averted relative to direct gaze. Biologically relevant stimuli such as facial expressions, gaze, or faces have been proposed to be processed by different pathways. An occipito-temporal visual pathway would be involved in the perceptual analysis of faces [112]. Another pathway, involving subcortical and cortical regions—including the amygdala, the pulvinar and the posterior parietal lobule—would subserve rapid, automatic (and potentially unconscious) orienting response to these stimuli [36, 89, 113–116]. This dual route view fits the two-process theory of face perception [117, 118], which was recently extended to eye contact detection [119]. This two-process theory proposes that distinct subcortical / cortical systems are involved in rapid detection and orienting response toward faces and direct gaze or eye contact (so-called Conspec process) on one hand and in perceptual analysis of faces (so-called Conlern) on the other hand. Our results may be interpreted in this framework: while the very early posterior parietal response would reflect the reflexive orienting response to direct gaze, the N170 amplitude modulation would reflect the perceptual analysis of gaze direction.

Some limitations to the present study should be considered. First, we only used pictures depicting frontal views of faces. This limits our conclusion to direct gaze under frontal view, which may bear particular low-level visual properties such as overall vertical symmetry. It would be interesting to use different head positions, as in past experiments [24, 42] to increase the generalization of our effect and test if it reflects true eye contact effects independent of head view. Moreover, in spite of the interpretation proposed above, the precise functional role of the early differential effect that we obtained remains to be identified. Only additional investigation, using for example transcranial magnetic stimulation (TMS) to disrupt the posterior parietal region in such early time range, will allow answering this question and determining if it can be used as a reliable marker of direct gaze detection. Besides, it would also be interesting to include a control condition such as closed eyes in order to tease apart the effects of direct and averted gazes. Indeed, it is well known that averted gaze can also trigger specific attention-related processes. We cannot exclude that the differentiated responses that we observed could be in part driven by the averted gaze condition. Finally, considering the very early latency of our effect, one may wonder if some electrical signal lingered in the electroretinogram and contributed to our source estimation, due to volume conduction. Although it cannot be ruled out, this however seems implausible because it is unclear how this could have resulted in the focal parietal activation obtained without any hint to additional activation in more anterior regions, near the eye balls.

In conclusion, our study confirms that gaze constitutes a highly relevant stimulus to which we are exquisitely sensitive. We demonstrated that gaze direction was detected early on by the human brain, with increased activity to direct versus averted gaze in the left posterior parietal region between 40 and 80 ms. This activity could provide a neural correlate of early attention capture by gaze contact.

## Supporting Information

S1 Fig Early ERP topographical pattern analysis in response to direct and averted gaze, in the high-spatial frequency (HSF) picture condition. Same legend as in Fig 4
(TIF)

S2 Fig Early ERP topographical pattern analysis in response to direct and averted gaze, in the low-spatial frequency (LSF) picture condition. Same legend as in Fig 4
(TIF)

## References

[1] Emery NJ. The eyes have it: The neuroethology, function and evolution of social gaze. Neuroscience and Biobehavioral Reviews. 2000;24(6):581–604. Epub 2000/08/15. . 10940436 10.1016/s0149-7634(00)00025-7

[2] Baron-Cohen S. Mindblindness: An essay on autism and theory of mind: MIT press; 1997.

[3] Friesen C, Kingstone A. The eyes have it! Reflexive orienting is triggered by nonpredictive gaze. Psychonomic Bulletin & Review. 1998;5(3):490–5.

[4] Frischen A, Bayliss AP, Tipper SP. Gaze cueing of attention: visual attention, social cognition, and individual differences. Psychological Bulletin. 2007;133(4):694–724. Epub 2007/06/27. 10.1037/0033-2909.133.4.694 17592962 PMC1950440

[5] George N, Conty L. Facing the gaze of others. Neurophysiologie Clinique-Clinical Neurophysiology. 2008;38(3):197–207. 10.1016/j.neucli.2008.03.001 . 18539254

[6] Kleinke CL. Gaze and eye contact: A research review. Psychological Bulletin. 1986;100(1):78–100. Epub 1986/07/01. . 3526377

[7] Conty L, Tijus C, Hugueville L, Coelho E, George N. Searching for asymmetries in the detection of gaze contact versus averted gaze under different head views: a behavioural study. Spatial vision. 2006;19(6):529–45. . 17278526 10.1163/156856806779194026

[8] Senju A, Hasegawa T. Do the upright eyes have it? Psychon Bull Rev. 2006;13(2):223–8. . 16892985 10.3758/bf03193834

[9] von Griinau M, Anston C. The detection of gaze direction: A stare-in-the-crowd effect. Perception. 1995;24(11):1297–313. 8643334 10.1068/p241297

[10] Stein T, Senju A, Peelen MV, Sterzer P. Eye contact facilitates awareness of faces during interocular suppression. Cognition. 2011;119(2):307–11. 10.1016/j.cognition.2011.01.008 21316650 PMC3796336

[11] Conty L, Gimmig D, Belletier C, George N, Huguet P. The cost of being watched: Stroop interference increases under concomitant eye contact. Cognition. 2010;115(1):133–9. 10.1016/j.cognition.2009.12.005 . 20070959

[12] Yokoyama T, Noguchi Y, Kita S. Unconscious processing of direct gaze: Evidence from an ERP study. Neuropsychologia. 2013;51(7):1161–8. 10.1016/j.neuropsychologia.2013.04.002 23603242

[13] Pourtois G, Thut G, Grave de Peralta R, Michel C, Vuilleumier P. Two electrophysiological stages of spatial orienting towards fearful faces: early temporo-parietal activation preceding gain control in extrastriate visual cortex. NeuroImage. 2005;26(1):149–63. 10.1016/j.neuroimage.2005.01.015 . 15862215

[14] Lehmann D, Skrandies W. Reference-free identification of components of checkerboard-evoked multichannel potential fields. Electroencephalogr Clin Neurophysiol. 1980;48(6):609–21. . 6155251 10.1016/0013-4694(80)90419-8

[15] Lachat F, Farroni T, George N. Watch Out! Magnetoencephalographic Evidence for Early Modulation of Attention Orienting by Fearful Gaze Cueing. Plos One. 2012;7(11). ARTN e50499 10.1371/journal.pone.0050499 . PMC3510181 23209761

[16] Bentin S, Allison T, Puce A, Perez E, McCarthy G. Electrophysiological studies of face perception in humans. Journal of Cognitive Neuroscience. 1996;8(6):551–65. 10.1162/jocn.1996.8.6.551 20740065 PMC2927138

[17] Rossion B, Jacques C. Does physical interstimulus variance account for early electrophysiological face sensitive responses in the human brain? Ten lessons on the N170. NeuroImage. 2008;39(4):1959–79. 10.1016/j.neuroimage.2007.10.011 . 18055223

[18] Itier R, Alain C, Kovacevic N, McIntosh A. Explicit versus implicit gaze processing assessed by ERPs. Brain Res. 2007;1177:79–89. 10.1016/j.brainres.2007.07.094 17916340

[19] Watanabe S, Miki K, Kakigi R. Gaze direction affects face perception in humans. Neurosci Lett. 2002;325(3):163–6. 12044646 10.1016/s0304-3940(02)00257-4

[20] Grice SJ, Halit H, Farroni T, Baron-Cohen S, Bolton P, Johnson MH. Neural correlates of eye-gaze detection in young children with autism. Cortex. 2005;41(3):342–53. 15871599 10.1016/s0010-9452(08)70271-5

[21] Klucharev V, Sams M. Interaction of gaze direction and facial expressions processing: ERP study. Neuroreport. 2004;15(4):621–5. 15094464 10.1097/00001756-200403220-00010

[22] Schweinberger SR, Kloth N, Jenkins R. Are you looking at me? Neural correlates of gaze adaptation. Neuroreport. 2007;18(7):693–6. 10.1097/WNR.0b013e3280c1e2d2 17426601

[23] Taylor MJ, Itier RJ, Allison T, Edmonds GE. Direction of gaze effects on early face processing: eyes-only versus full faces. Cognitive Brain Research. 2001;10(3):333–40. 11167057 10.1016/s0926-6410(00)00051-3

[24] Conty L, N'Diaye K, Tijus C, George N. When eye creates the contact! ERP evidence for early dissociation between direct and averted gaze motion processing. Neuropsychologia. 2007;45(13):3024–37. 10.1016/j.neuropsychologia.2007.05.017 . 17644145

[25] Latinus M, Love SA, Rossi A, Parada F, Huang L, Conty L, et al Social decisions affect neural activity to perceived dynamic gaze. Soc Cogn Affect Neurosci. 2015 10.1093/scan/nsv049 . 25925272 PMC4631155

[26] Taylor MJ, George N, Ducorps A. Magnetoencephalographic evidence of early processing of direction of gaze in humans. Neurosci Lett. 2001;316(3):173–7. . 11744230 10.1016/s0304-3940(01)02378-3

[27] Watanabe S, Kakigi R, Miki K, Puce A. Human MT/V5 activity on viewing eye gaze changes in others: A magnetoencephalographic study. Brain Res. 2006;1092:152–60. 10.1016/j.brainres.2006.03.091 . 16684514

[28] Michel CM, Thut G, Morand S, Khateb A, Pegna AJ, Grave de Peralta R, et al Electric source imaging of human brain functions. Brain research Brain research reviews. 2001;36(2–3):108–18. . 11690607 10.1016/s0165-0173(01)00086-8

[29] Picton TW, Bentin S, Berg P, Donchin E, Hillyard SA, Johnson R Jr., et al Guidelines for using human event-related potentials to study cognition: recording standards and publication criteria. Psychophysiology. 2000;37(2):127–52. . 10731765

[30] Caldara R, Rossion B, Bovet P, Hauert CA. Event-related potentials and time course of the "other-race" face classification advantage. Neuroreport. 2004;15(5):905–10. . 15073540 10.1097/00001756-200404090-00034

[31] Itier R, Taylor M. N170 or N1? Spatiotemporal differences between object and face processing using ERPs. Cerebral Cortex. 2004;14(2):132–42. 14704210 10.1093/cercor/bhg111

[32] Latinus M, Taylor MJ. Face processing stages: impact of difficulty and the separation of effects. Brain Res. 2006;1123(1):179–87. 10.1016/j.brainres.2006.09.031 . 17054923

[33] Morand SM, Harvey M, Grosbras MH. Parieto-occipital cortex shows early target selection to faces in a reflexive orienting task. Cereb Cortex. 2014;24(4):898–907. 10.1093/cercor/bhs368 . 23183710

[34] Pizzagalli D, Lehmann D, Koenig T, Regard M, Pascual-Marqui RD. Face-elicited ERPs and affective attitude: brain electric microstate and tomography analyses. Clinical neurophysiology: official journal of the International Federation of Clinical Neurophysiology. 2000;111(3):521–31. . 10699416 10.1016/s1388-2457(99)00252-7

[35] Thierry G, Martin CD, Downing P, Pegna AJ. Controlling for interstimulus perceptual variance abolishes N170 face selectivity. Nature neuroscience. 2007;10(4):505–11. 10.1038/nn1864 . 17334361

[36] Senju A, Johnson MH. The eye contact effect: Mechanisms and development. Trends Cogn Sci. 2009;13(3):127–34. Epub 2009/02/17. 10.1016/j.tics.2008.11.009 . 19217822

[37] Bar M. A cortical mechanism for triggering top-down facilitation in visual object recognition. Journal of Cognitive Neuroscience. 2003;15(4):600–9. 10.1162/089892903321662976 . 12803970

[38] Bullier J, Nowak LG. Parallel Versus Serial Processing—New Vistas on the Distributed Organization of the Visual-System. Curr Opin Neurobiol. 1995;5(4):497–503. 10.1016/0959-4388(95)80011-5 . 7488852

[39] Merigan WH, Maunsell JHR. How parallel are the primate visual pathways. Annu Rev Neurosci. 1993;16:369–402. 10.1146/annurev.ne.16.030193.002101 . 8460898

[40] Shapley R. Visual sensitivity and parallel retinocortical channels. Annu Rev Psychol. 1990;41:635–58. 10.1146/annurev.psych.41.1.635 . 2407178

[41] Adams RB Jr., Kleck RE. Effects of direct and averted gaze on the perception of facially communicated emotion. Emotion. 2005;5(1):3–11. Epub 2005/03/10. 10.1037/1528-3542.5.1.3 . 15755215

[42] George N, Driver J, Dolan RJ. Seen gaze-direction modulates fusiform activity and its coupling with other brain areas during face processing. NeuroImage. 2001;13(6 Pt 1):1102–12. Epub 2001/05/16. 10.1006/nimg.2001.0769 . 11352615

[43] Kawashima R, Sugiura M, Kato T, Nakamura A, Hatano K, Ito K, et al The human amygdala plays an important role in gaze monitoring. A PET study. Brain. 1999;122 (Pt 4):779–83. Epub 1999/04/29. . 10219788 10.1093/brain/122.4.779

[44] Mosher CP, Zimmerman PE, Gothard KM. Neurons in the monkey amygdala detect eye contact during naturalistic social interactions. Current Biology. 2014;24(20):2459–64. 10.1016/j.cub.2014.08.063 25283782 PMC4253056

[45] Burra N, Hervais-Adelman A, Kerzel D, Tamietto M, de Gelder B, Pegna AJ. Amygdala activation for eye contact despite complete cortical blindness. J Neurosci. 2013;33(25):10483–9. 10.1523/JNEUROSCI.3994-12.2013 . 23785160 PMC6618602

[46] Mormann F, Niediek J, Tudusciuc O, Quesada CM, Coenen VA, Elger CE, et al Neurons in the human amygdala encode face identity, but not gaze direction. Nat Neurosci. 2015;18(11):1568–70. 10.1038/nn.4139 26479589 PMC4624486

[47] Delplanque S, N'Diaye K, Scherer K, Grandjean D. Spatial frequencies or emotional effects? A systematic measure of spatial frequencies for IAPS pictures by a discrete wavelet analysis. Journal of Neuroscience Methods. 2007;165(1):144–50. Epub 2007/07/17. 10.1016/j.jneumeth.2007.05.030 . 17629569

[48] Acunzo DJ, MacKenzie G, van Rossum MC. Systematic biases in early ERP and ERF components as a result of high-pass filtering. Journal of Neuroscience Methods. 2012;209(1):212–8. 10.1016/j.jneumeth.2012.06.011 22743800

[49] Perrin F, Pernier J, Bertrand O, Echallier J. Spherical splines for scalp potential and current density mapping. Electroencephalography and clinical Neurophysiology. 1989;72(2):184–7. 2464490 10.1016/0013-4694(89)90180-6

[50] Heisz JJ, Watter S, Shedden JM. Progressive N170 habituation to unattended repeated faces. Vision Research. 2006;46(1):47–56. 16289274 10.1016/j.visres.2005.09.028

[51] Vizioli L, Rousselet GA, Caldara R. Neural repetition suppression to identity is abolished by other-race faces. Proceedings of the National Academy of Sciences. 2010;107(46):20081–6. 10.1073/pnas.1005751107 PMC2993371 21041643

[52] Brunet D, Murray MM, Michel CM. Spatiotemporal analysis of multichannel EEG: CARTOOL. Computational intelligence and neuroscience. 2011;2011:813870 10.1155/2011/813870 21253358 PMC3022183

[53] Murray MM, Brunet D, Michel CM. Topographic ERP analyses: a step-by-step tutorial review. Brain topography. 2008;20(4):249–64. 10.1007/s10548-008-0054-5 18347966

[54] Pourtois G, Delplanque S, Michel C, Vuilleumier P. Beyond conventional event-related brain potential (ERP): exploring the time-course of visual emotion processing using topographic and principal component analyses. Brain topography. 2008;20(4):265–77. 10.1007/s10548-008-0053-6 . 18338243

[55] Michel CM, Lantz G, Spinelli L, De Peralta RG, Landis T, Seeck M. 128-channel EEG source imaging in epilepsy: clinical yield and localization precision. Journal of clinical neurophysiology: official publication of the American Electroencephalographic Society. 2004;21(2):71–83. . 15284597 10.1097/00004691-200403000-00001

[56] Michel CM, Murray MM. Towards the utilization of EEG as a brain imaging tool. NeuroImage. 2012;61(2):371–85. 10.1016/j.neuroimage.2011.12.039 . 22227136

[57] Michel CM, Murray MM, Lantz G, Gonzalez S, Spinelli L, Grave de Peralta R. EEG source imaging. Clinical Neurophysiology. 2004;115(10):2195–222. 10.1016/j.clinph.2004.06.001 . 15351361

[58] Pascual-Marqui RD, Michel CM, Lehmann D. Segmentation of brain electrical activity into microstates: model estimation and validation. IEEE transactions on bio-medical engineering. 1995;42(7):658–65. 10.1109/10.391164 . 7622149

[59] Lehmann D, Ozaki H, Pal I. EEG alpha map series: brain micro-states by space-oriented adaptive segmentation. Electroencephalogr Clin Neurophysiol. 1987;67(3):271–88. . 2441961 10.1016/0013-4694(87)90025-3

[60] Seeck M, Michel CM, Mainwaring N, Cosgrove R, Blume H, Ives J, et al Evidence for rapid face recognition from human scalp and intracranial electrodes. Neuroreport. 1997;8(12):2749–54. . 9295112 10.1097/00001756-199708180-00021

[61] Gianotti LR, Faber PL, Schuler M, Pascual-Marqui RD, Kochi K, Lehmann D. First valence, then arousal: the temporal dynamics of brain electric activity evoked by emotional stimuli. Brain topography. 2008;20(3):143–56. 10.1007/s10548-007-0041-2 . 18175212

[62] Pourtois G, Dan ES, Grandjean D, Sander D, Vuilleumier P. Enhanced extrastriate visual response to bandpass spatial frequency filtered fearful faces: Time course and topographic evoked-potentials mapping. Hum Brain Mapp. 2005;26(1):65–79. 10.1002/Hbm.20130 . 15954123 PMC6871777

[63] Michel CM, Grave de Peralta R, Lantz G, Gonzalez Andino S, Spinelli L, Blanke O, et al Spatiotemporal EEG analysis and distributed source estimation in presurgical epilepsy evaluation. Journal of clinical neurophysiology: official publication of the American Electroencephalographic Society. 1999;16(3):239–66. . 10426407 10.1097/00004691-199905000-00005

[64] Grave-de Peralta R, Gonzalez-Andino S, Gomez-Gonzalez CM. [The biophysical foundations of the localisation of encephalogram generators in the brain. The application of a distribution-type model to the localisation of epileptic foci]. Revista de neurologia. 2004;39(8):748–56. . 15514904

[65] Spinelli L, Andino SG, Lantz G, Seeck M, Michel CM. Electromagnetic inverse solutions in anatomically constrained spherical head models. Brain topography. 2000;13(2):115–25. . 11154101 10.1023/a:1026607118642

[66] Ary JP, Klein SA, Fender DH. Location of sources of evoked scalp potentials: corrections for skull and scalp thicknesses. IEEE transactions on bio-medical engineering. 1981;28(6):447–52. 10.1109/TBME.1981.324817 . 7287042

[67] De Lucia M, Clarke S, Murray MM. A temporal hierarchy for conspecific vocalization discrimination in humans. J Neurosci. 2010;30(33):11210–21. 10.1523/JNEUROSCI.2239-10.2010 . 20720129 PMC6633490

[68] Knebel JF, Murray MM. Towards a resolution of conflicting models of illusory contour processing in humans. Neuroimage. 2012;59(3):2808–17. 10.1016/j.neuroimage.2011.09.031 . 21979384

[69] West GL, Anderson AA, Ferber S, Pratt J. Electrophysiological evidence for biased competition in V1 for fear expressions. Journal of Cognitive Neuroscience. 2011;23(11):3410–8. 10.1162/jocn.2011.21605 . 21281089

[70] Zhu XR, Luo YJ. Fearful faces evoke a larger C1 than happy faces in executive attention task: an event-related potential study. Neurosci Lett. 2012;526(2):118–21. 10.1016/j.neulet.2012.08.011 22910608 PMC3632666

[71] Culham JC, Brandt SA, Cavanagh P, Kanwisher NG, Dale AM, Tootell RB. Cortical fMRI activation produced by attentive tracking of moving targets. Journal of Neurophysiology. 1998;80(5):2657–70. 9819271 10.1152/jn.1998.80.5.2657

[72] Langton SR, Bruce V. Reflexive visual orienting in response to the social attention of others. Visual Cognition. 1999;6(5):541–67.

[73] Simon O, Mangin J-F, Cohen L, Le Bihan D, Dehaene S. Topographical layout of hand, eye, calculation, and language-related areas in the human parietal lobe. Neuron. 2002;33(3):475–87. 11832233 10.1016/s0896-6273(02)00575-5

[74] Le TH, Pardo JV, Hu X. 4 T-fMRI study of nonspatial shifting of selective attention: cerebellar and parietal contributions. Journal of Neurophysiology. 1998;79(3):1535–48. 9497430 10.1152/jn.1998.79.3.1535

[75] Behrmann M, Geng JJ, Shomstein S. Parietal cortex and attention. Curr Opin Neurobiol. 2004;14(2):212–7. 10.1016/j.conb.2004.03.012 . 15082327

[76] Ohman A. Face the beast and fear the face: animal and social fears as prototypes for evolutionary analyses of emotion. Psychophysiology. 1986;23(2):123–45. . 3704069 10.1111/j.1469-8986.1986.tb00608.x

[77] Vuilleumier P. How brains beware: neural mechanisms of emotional attention. Trends Cogn Sci. 2005;9(12):585–94. 10.1016/j.tics.2005.10.011 . 16289871

[78] Margulies DS, Vincent JL, Kelly C, Lohmann G, Uddin LQ, Biswal BB, et al Precuneus shares intrinsic functional architecture in humans and monkeys. Proc Natl Acad Sci U S A. 2009;106(47):20069–74. 10.1073/pnas.0905314106 . 19903877 PMC2775700

[79] Zhang S, Li CSR. Functional connectivity mapping of the human precuneus by resting state fMRI. Neuroimage. 2012;59(4):3548–62. 10.1016/j.neuroimage.2011.11.023 . 22116037 PMC3288461

[80] Balan PF, Gottlieb J. Integration of exogenous input into a dynamic salience map revealed by perturbing attention. The Journal of Neuroscience. 2006;26(36):9239–49. 10.1523/JNEUROSCI.1898-06.2006 16957080 PMC6674497

[81] Bendiksby MS, Platt ML. Neural correlates of reward and attention in macaque area LIP. Neuropsychologia. 2006;44(12):2411–20. 10.1016/j.neuropsychologia.2006.04.011 16757005

[82] Fecteau JH, Munoz DP. Salience, relevance, and firing: a priority map for target selection. Trends Cogn Sci. 2006;10(8):382–90. 10.1016/j.tics.2006.06.011 16843702

[83] Geng JJ, Mangun GR. Anterior intraparietal sulcus is sensitive to bottom–up attention driven by stimulus salience. Journal of Cognitive Neuroscience. 2009;21(8):1584–601. 10.1162/jocn.2009.21103 18752405

[84] Zenon A, Filali N, Duhamel J-R, Olivier E. Salience representation in the parietal and frontal cortex. Journal of Cognitive Neuroscience. 2010;22(5):918–30. 10.1162/jocn.2009.21233 19366288

[85] Macrae CN, Hood BM, Milne AB, Rowe AC, Mason MF. Are you looking at me? Eye gaze and person perception. Psychological Science. 2002;13(5):460–4. 12219814 10.1111/1467-9280.00481

[86] Radua J, Phillips ML, Russell T, Lawrence N, Marshall N, Kalidindi S, et al Neural response to specific components of fearful faces in healthy and schizophrenic adults. NeuroImage. 2010;49(1):939–46. 10.1016/j.neuroimage.2009.08.030 19699306

[87] Bonda E, Petrides M, Ostry D, Evans A. Specific involvement of human parietal systems and the amygdala in the perception of biological motion. The Journal of Neuroscience. 1996;16(11):3737–44. 8642416 10.1523/JNEUROSCI.16-11-03737.1996 PMC6578830

[88] Anders S, Birbaumer N, Sadowski B, Erb M, Mader I, Grodd W, et al Parietal somatosensory association cortex mediates affective blindsight. Nature neuroscience. 2004;7(4):339–40. 10.1038/nn1213 . 15034586

[89] Troiani V, Price ET, Schultz RT. Unseen fearful faces promote amygdala guidance of attention. Social Cognitive and Affective Neuroscience. 2014;9(2):133–40. 10.1093/scan/nss116 23051897 PMC3907921

[90] Kobayashi H, Kohshima S. Unique morphology of the human eye. Nature. 1997;387(6635):767–8. Epub 1997/06/19. 10.1038/42842 . 9194557

[91] Bullier J. Integrated model of visual processing. Brain research Brain research reviews. 2001;36(2–3):96–107. . 11690606 10.1016/s0165-0173(01)00085-6

[92] Hamm AO, Weike AI, Schupp HT, Treig T, Dressel A, Kessler C. Affective blindsight: intact fear conditioning to a visual cue in a cortically blind patient. Brain. 2003;126(Pt 2):267–75. . 12538396 10.1093/brain/awg037

[93] Foxe JJ, Simpson GV. Flow of activation from V1 to frontal cortex in humans. A framework for defining "early" visual processing. Exp Brain Res. 2002;142(1):139–50. 10.1007/s00221-001-0906-7 . 11797091

[94] Schroeder CE, Mehta AD, Givre SJ. A spatiotemporal profile of visual system activation revealed by current source density analysis in the awake macaque. Cereb Cortex. 1998;8(7):575–92. . 9823479 10.1093/cercor/8.7.575

[95] Yoshor D, Ghose GM, Bosking WH, Sun P, Maunsell JH. Spatial attention does not strongly modulate neuronal responses in early human visual cortex. J Neurosci. 2007;27(48):13205–9. 10.1523/JNEUROSCI.2944-07.2007 . 18045914 PMC6673408

[96] Ouellette BG, Casanova C. Overlapping visual response latency distributions in visual cortices and LP-pulvinar complex of the cat. Exp Brain Res. 2006;175(2):332–41. 10.1007/s00221-006-0555-y . 16816944

[97] Sugase Y, Yamane S, Ueno S, Kawano K. Global and fine information coded by single neurons in the temporal visual cortex. Nature. 1999;400(6747):869–73. 10.1038/23703 . 10476965

[98] Maior RS, Hori E, Tomaz C, Ono T, Nishijo H. The monkey pulvinar neurons differentially respond to emotional expressions of human faces. Behav Brain Res. 2010;215(1):129–35. 10.1016/j.bbr.2010.07.009 . 20643164

[99] Clower DM, West RA, Lynch JC, Strick PL. The inferior parietal lobule is the target of output from the superior colliculus, hippocampus, and cerebellum. J Neurosci. 2001;21(16):6283–91. . 11487651 10.1523/JNEUROSCI.21-16-06283.2001 PMC6763148

[100] Asanuma C, Andersen RA, Cowan WM. The thalamic relations of the caudal inferior parietal lobule and the lateral prefrontal cortex in monkeys: divergent cortical projections from cell clusters in the medial pulvinar nucleus. J Comp Neurol. 1985;241(3):357–81. 10.1002/cne.902410309 . 4086661

[101] Hardy SG, Lynch JC. The spatial distribution of pulvinar neurons that project to two subregions of the inferior parietal lobule in the macaque. Cereb Cortex. 1992;2(3):217–30. . 1511222 10.1093/cercor/2.3.217

[102] Baizer JS, Maguire WM. Double representation of lower visual quadrant in prelunate gyrus of rhesus monkey. Invest Ophthalmol Vis Sci. 1983;24(10):1436–9. . 6618807

[103] Di Russo F, Martínez A, Hillyard SA. Source analysis of event-related cortical activity during visuo-spatial attention. Cerebral Cortex. 2003;13(5):486–99. 12679295 10.1093/cercor/13.5.486

[104] Mangun GR, Hillyard SA, Luck SJ. IQ Electrocortical substrates of visual selective attention. Attention and performance XIV: Synergies in experimental psychology, artificial intelligence, and cognitive neuroscience. 1993;14:219.

[105] Martinez A, Anllo-Vento L, Sereno MI, Frank LR, Buxton RB, Dubowitz D, et al Involvement of striate and extrastriate visual cortical areas in spatial attention. Nature neuroscience. 1999;2(4):364–9. 10.1038/7274 10204544

[106] Collin CA, Therrien ME, Campbell KB, Hamm JP. Effects of band-pass spatial frequency filtering of face and object images on the amplitude of N170. Perception. 2012;41(6):717–32. . 23094460 10.1068/p7056

[107] Flevaris AV, Robertson LC, Bentin S. Using spatial frequency scales for processing face features and face configuration: an ERP analysis. Brain Res. 2008;1194:100–9. 10.1016/j.brainres.2007.11.071 . 18190897

[108] Perrett DI, Oram MW, Ashbridge E. Evidence accumulation in cell populations responsive to faces: an account of generalisation of recognition without mental transformations. Cognition. 1998;67(1–2):111–45. . 9735538 10.1016/s0010-0277(98)00015-8

[109] Goffaux V, Gauthier I, Rossion B. Spatial scale contribution to early visual differences between face and object processing. Brain Res Cogn Brain Res. 2003;16(3):416–24. . 12706221 10.1016/s0926-6410(03)00056-9

[110] Hung Y, Smith ML, Bayle DJ, Mills T, Cheyne D, Taylor MJ. Unattended emotional faces elicit early lateralized amygdala-frontal and fusiform activations. Neuroimage. 2010;50(2):727–33. 10.1016/j.neuroimage.2009.12.093 . 20045736

[111] Ulloa JL, Puce A, Hugueville L, George N. Sustained neural activity to gaze and emotion perception in dynamic social scenes. Social Cognitive and Affective Neuroscience. 2014;9(3):350–7. 10.1093/scan/nss141 23202662 PMC3980798

[112] Haxby JV, Hoffman EA, Gobbini MI. The distributed human neural system for face perception. Trends Cogn Sci. 2000;4(6):223–33. 10827445 10.1016/s1364-6613(00)01482-0

[113] Pessoa L, Adolphs R. Emotion processing and the amygdala: from a 'low road' to 'many roads' of evaluating biological significance. Nat Rev Neurosci. 2010;11(11):773–83. 10.1038/nrn2920 20959860 PMC3025529

[114] Bauer RM. Autonomic recognition of names and faces in prosopagnosia: a neuropsychological application of the Guilty Knowledge Test. Neuropsychologia. 1984;22(4):457–69. . 6483172 10.1016/0028-3932(84)90040-x

[115] Bear DM. Hemispheric specialization and the neurology of emotion. Archives of Neurology. 1983;40(4):195 6830468 10.1001/archneur.1983.04050040025003

[116] Ellis HD, Young AW. Accounting for delusional misidentifications. Br J Psychiatry. 1990;157:239–48. . 2224375 10.1192/bjp.157.2.239

[117] Johnson M, Morton J. Biology and cognitive development: The case of face recognitionBlackwell. New York. 1991.

[118] Morton J, Johnson MH. CONSPEC and CONLERN: a two-process theory of infant face recognition. Psychological Review. 1991;98(2):164 2047512 10.1037/0033-295x.98.2.164

[119] Johnson M, Senju A, Tomalski P. The two-process theory of face processing: Modifications based on two decades of data from infants and adults. Neurosci Biobehav Rev. 2015;50C:169–79. 10.1016/j.neubiorev.2014.10.009 . 25454353

