# Supplementary figures and images for: Early Left Parietal Activity Elicited by Direct Gaze: A High-Density EEG Study
Source: PLoS One. 2016 Nov 23;11(11):e0166430. doi: 10.1371/journal.pone.0166430 (PMC5120811; doi:10.1371/journal.pone.0166430)

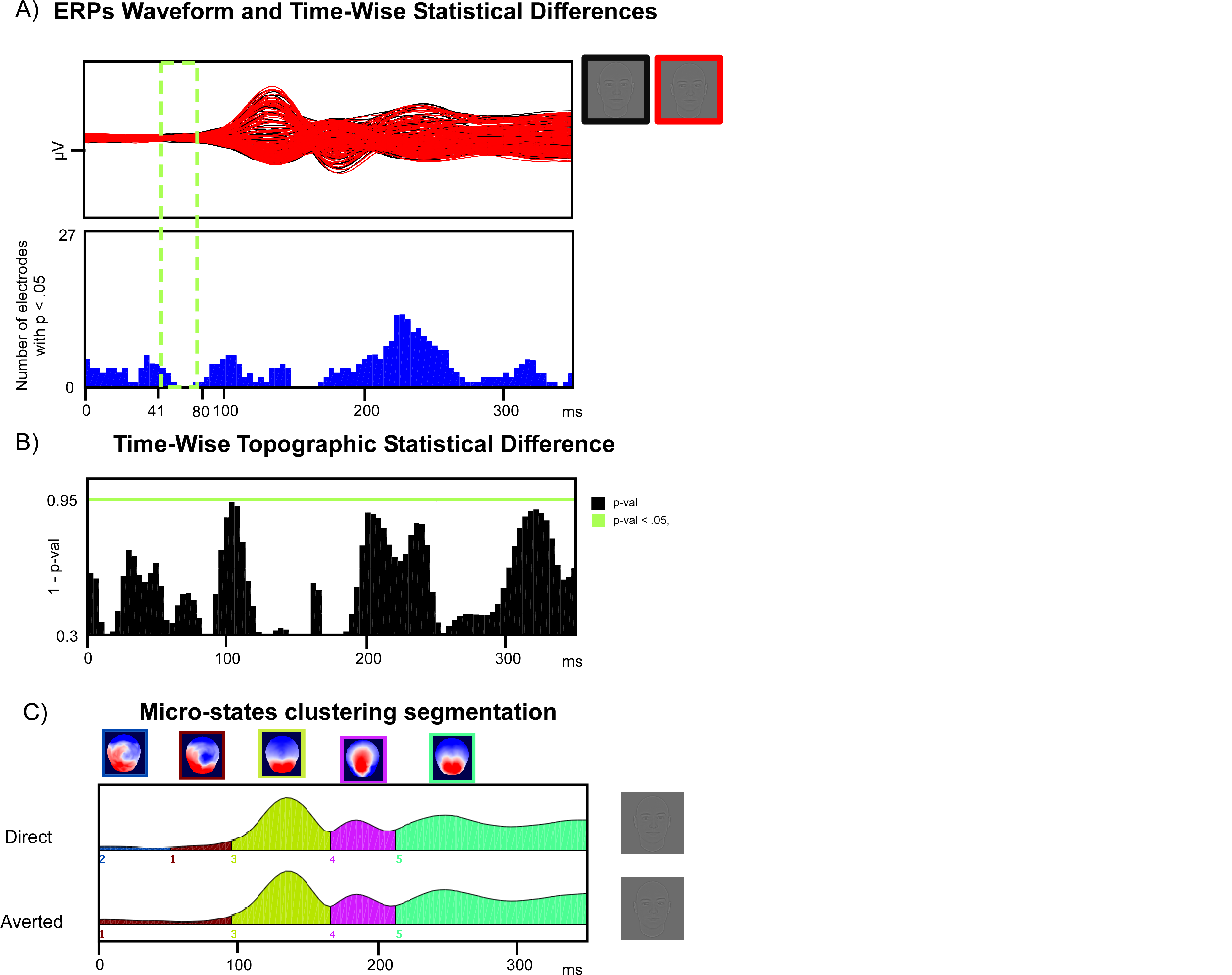

Supplement: S1 Fig — Same legend as in Fig 4 (TIF) [file pone.0166430.s001.tif]

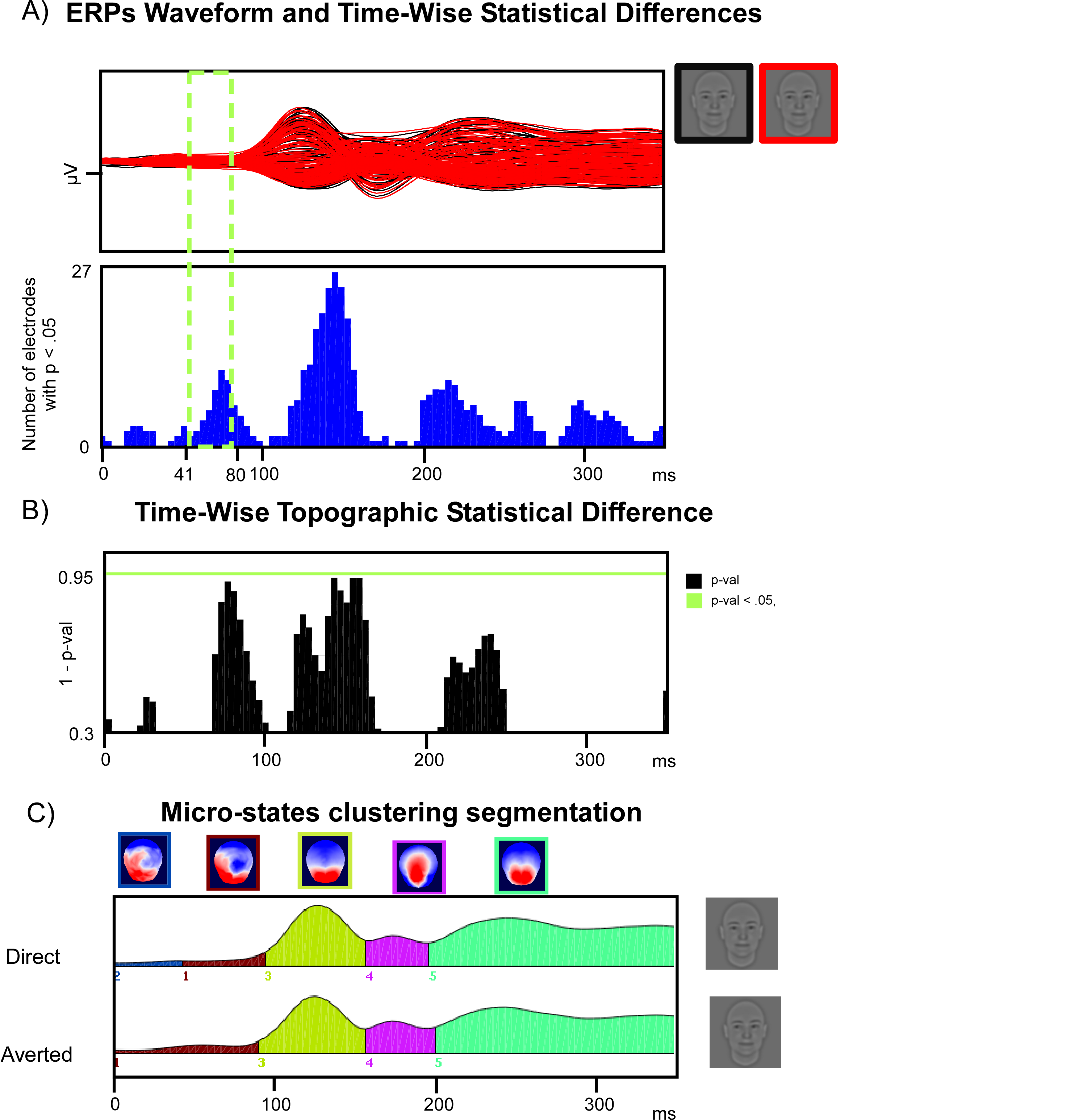

Supplement: S2 Fig — Same legend as in Fig 4 (TIF) [file pone.0166430.s002.tif]
